# Communication partner training for student health and social care professionals engaging with people with stroke acquired communication difficulties: A protocol for a realist review.

**DOI:** 10.12688/hrbopenres.13783.2

**Published:** 2024-04-17

**Authors:** Yvonne Fitzmaurice, Suzanne Beeke, Jytte Isaksen, Una Cunningham, Caroline Jagoe, Éidín Ní Shé, Ruth McMenamin

**Affiliations:** 1 School of Health Sciences, University of Galway, Galway, H91 TK33, Ireland; 2 Division of Psychology and Language Sciences, University College London, London, England, WC1E 6BT, UK; 3 Department of Language, Culture, History and Communication, University of Souhern Denmark, Odense, Denmark; 4 Mater Misericordiae University Hospital, Dublin, D07 R2WY, Ireland; 5 School of Linguistics, Speech and Communication Sciences, The University of Dublin Trinity College, Dublin, Leinster, D02 PN40, Ireland; 6 Speech Pathology and Audiology, School of Human and Communication Development, University of Witwatersrand, Johannesburg, South Africa; 7 Graduate School of Healthcare Management, RCSI University of Medicine and Health Sciences, Dublin, D02 YN77, Ireland; 8 PPI Ignite Network @ University of Galway, University of Galway, Galway, H91 TK33, Ireland

**Keywords:** Communication partner training, student health and social care professionals, stroke, aphasia, acquired apraxia of speech, dysarthria, cognitive communication disorder, public and patient involvement

## Abstract

**Background:**

Stroke acquired communication impairments impede effective communication. Consequently, in stroke care, communicative interactions can be challenging for both patients and staff and can predispose patients to increased risk of preventable adverse events. Communication partner training (CPT) can mitigate such negative outcomes by optimising communicative interactions. Providing CPT to student health and social care professionals (SH&SCPs) has the potential to enhance their clinical expertise and experiences and enhance the future clinical care of patients with stroke acquired communication impairments. This research aims to expand our understanding of how CPT is operationalised for SH&SCPs in higher education institutions and determine: what works; for whom; in what contexts; how and why?

**Methods:**

This review is Phase 1 of a research project employing a realist approach with public and patient involvement (PPI). It incorporates five iterative steps: 1.) Clarifying the scope; 2.) Searching for evidence; 3.) Selecting and appraising evidence; 4.) Data extraction; 5.) Synthesising data and developing a middle range theory explaining how CPT is expected to work for SH&SCPs. An advisory panel, including PPI advisors, content advisors, student advisors, realist advisors and educationalist advisor has been set up to consult throughout the review and collaboratively agree the middle range theory.

**Discussion:**

While there is an evolving evidence base for CPT, including stroke specific CPT for SH&SCPs, it is acknowledged that there are challenges to its implementation in complex real-world settings. In combining empirical evidence with theoretical understanding, realist review permits synthesis of data from diverse sources and goes beyond determining efficacy to explore generative causation and solutions for real world practice. A middle range realist programme theory that coherently explains how CPT is expected to work when teaching SH&SCPs to communicate with people with stroke acquired communication impairments will provide educators with new insights into CPT development and implementation in their higher education institutions.

## Abbreviations

CPT: Communication partner training; SH&SCPs: Student health and social care professionals; IPT/s: Initial programme theory/theories; C: Context; M: Mechanism; O: Outcome; CMOC: Context, mechanism, outcome configuration; PPI: Public and Patient Involvement; TDF: Theoretical Domains Framework; BCW: Behaviour Change Wheel

## Introduction

### Stroke acquired communication impairments

In 2019 the global incidence of stroke was reported to be 12.2 million and stroke was reported as the third leading cause of death and disability resulting in 143 million people living with “disability-adjusted life-years" ( Stark *et al.,* 2021, p. 795). Among these life adjusting disabilities are the communication impairments aphasia, dysarthria, apraxia of speech and cognitive communication disorders ( Baker *et al.,* 2022). These impairments (overviewed in Table 1) can occur in isolation or in varying combinations and with varying severities. Their incidence and prevalence will increase in the coming decades in line with the predicted increased incidence of stroke world-wide ( Stark *et al.,* 2021). Specific to the European Union, Wafa *et al.* (2020) anticipate that the people living with stroke will have increased by 27% between 2017 and 2047. Studies reporting on the specific incidence and prevalence of stroke acquired communication impairments are variable, for example, they differ in geographical origins, number of participants and designs and methods ( Frederick *et al.,* 2022; Mitchell *et al.,* 2021). Hence, there are variations across reports with aphasia impacting 7% to 77%, dysarthria impacting 24% to 69% and a combination of dysarthria and aphasia impacting 4% to 29% ( Ali *et al.,* 2015; De Cock *et al.,* 2020; Dickey *et al.,* 2010; Flowers *et al.,* 2013; Frederick *et al.,* 2022; Mitchell *et al.,* 2021; Stipancic *et al.,* 2019). Data on incidence of stroke acquired apraxia of speech is sparse and challenging to report on given that acquired apraxia of speech rarely exists in isolations but typically co-occurs with aphasia and/or dysarthria ( Duffy, 2020). Regarding cognitive communication disorders associated with acute stroke, Riepe *et al.* (2004) reported that up to 77% exhibited cognitive impairment.

**Table 1.** Overview of stroke acquired communication impairments.

Impairment	Common clinical manifestations
**Aphasia**	Aphasia can impair an individual’s ability to express themselves; understand what others are saying or read and write. In mild cases there can be word finding difficulties and difficulty understanding complex written and/or verbal instructions. In severe cases, it may be difficult to verbalise, write or understand even single words.
**Dysarthria**	Dysarthria, an umbrella term for a group of speech disorders, reflects abnormalities in strength, speed, range, steadiness, tone, or accuracy of movements required for speech production ( Duffy, 2020, p. 3). Intelligibility of speech is impacted. Speech may be imprecise, slow, monotonous or low in volume. In mild cases an individual may have to occasionally repeat themselves to be understood. In severe cases speech may be entirely unintelligible.
**Acquired Apraxia of** **Speech**	Typically an individual knows what they want to say, but cannot produce and sequence sounds correctly in words. Speech can be slow and monotonous with reduced intelligibility. Automatic speech is better preserved than volitional speech. In mild cases intelligibility may be minimally impaired. In severe cases an individual may have difficulty producing any sounds or words. Acquired apraxia of speech rarely occurs in isolation, but co-exists with aphasia or dysarthria.
**Cognitive** **Communication** **Disorder**	Cognitive communication difficulties result from impairment to underlying cognitive functions such as memory, executive function, and attention. They have variable presentations and can include difficulties turn-taking in conversation; staying on topic; attending to and recalling specific details and giving information in the correct sequence.

The stroke acquired communication impairments outlined in Table 1 present chronic, multifaceted challenges for the individuals presenting with the impairment/s and for those interacting with them ( Ali *et al.,* 2015; Chang *et al.,* 2018; Mitchell *et al.,* 2021; Wray *et al.,* 2019). They can mask the individual’s inherent competency and result in communicative interactions that are negative for all interlocutors ( Carragher *et al.,* 2020; Kagan *et al.,* 2001; Kagan *et al.,* 2018; van Rijssen *et al.,* 2021). Unsuccessful communication predisposes patients to increased risk of adverse hospital events ( Bartlett *et al.,* 2008; Hemsley *et al.,* 2013); loss of autonomy and exclusion from participation in care related decisions ( Brady *et al.,* 2011; Carragher *et al.,* 2020; O’Halloran *et al.,* 2012). Additionally, there is increased risk of depression and anxiety ( Shehata *et al.,* 2015; Zanella *et al.,* 2023).

When communication with patients is challenging, health and social care professionals (i.e., professionals with formal clinical training, e.g., doctors, nurses, physiotherapists, psychologists) can experience emotions such as frustration, impatience and guilt ( Carragher *et al.,* 2020; Hur & Kang, 2022). Consequently, they can limit their time interacting with people with communication impairments ( Carragher *et al.,* 2020). Student health and social care professionals (SH&SCPs) who may also, in contexts where professional registration is required, be referred to as pre-registration professionals, experience similar emotions. Over the course of their clinical training these students may be exposed to a variety of pedagogies and engage in a range of clinical activities and placements involving people with stroke acquired communication difficulties. Rathiram *et al.* (2022, p. 5) reported that SH&SCPs felt “emotionally strained, frustrated and helpless” when they could not understand their patients. In order to prevent these negative experiences health and social care professionals and SH&SCPs want to learn how to communicate successfully with people with communication difficulties ( Carragher *et al.,* 2020; Hur & Kang, 2022). Furthermore, people with aphasia believe that training health care professionals in the use of communication strategies is important in enabling them to live successfully with the communication impairment ( Manning *et al.,* 2019). CPT can provide the necessary learning opportunities.

### Communication Partner Training (CPT)

CPT is defined by Cruice *et al.* (2018, p. 1) as an “umbrella term for a complex, behavioural intervention” that has many interacting components that are delivered in flexible ways. It is also described as an environmental intervention ( Simmons-Mackie *et al.,* 2010; Simmons-Mackie *et al.,* 2016) as people around the person with communication impairment/s ( *i.e.,* the communication partners) adjust their behaviour and use communicative strategies and resources to facilitate information exchange ( Simmons-Mackie *et al.,* 2010; Simmons-Mackie *et al.,* 2016). Such strategies and resources may include non-verbal cues such as gesture and pointing; personally relevant communication books and folders; applications on smart devices; writing; visual aids ( *e.g.,* maps, photo diaries) and modified verbal expression ( *e.g.,* slowed rate, using key words and short phrases). CPT can be successfully employed with multiple communication partners including health and social care professionals, SH&SCPs, family members, volunteers and people with communication impairments ( Beeke *et al.,* 2018; Cameron *et al.,* 2018; Forsgren *et al.,* 2017; Isaksen *et al.,* 2023; Kagan *et al.,* 2001; Rayner & Marshall, 2003). Importantly, CPT has the potential to pre-empt communicative challenges for our future frontline staff and enhance future clinical care for patients with stroke acquired communication impairments.

### Employing realist review in CPT research

Realist terminology relevant to this realist review and all other phases of this research project is presented in Table 2 below.

**Table 2.** Explanation of realist terminology employed across all phases of research project.

Concept	Explanation
**Context (C)**	“Any condition that triggers and/or modifies the behaviour of a mechanism” in the generation of outcomes ( Duddy & Wong, 2023, p. 3). Contexts may be social, psychological, material, organisational, economic etc. ( http://www. ramesesproject.org/media/RAMESES_II_Context.pdf).
**Mechanisms** **(M)**	Underlying entities, forces or processes which operate in particular contexts to enact outcomes ( Astbury & Leeuw, 2010). They change the “reasoning and responses” of individuals to enact outcomes ( Dalkin *et al.,* 2015) and are frequently hidden ( Jagosh, 2019).
**Outcome (O)**	The consequence of the intervention. May be visible, measurable, proximal, distal, intended and/or unintended ( Jagosh *et al.,* 2014).
**Context,** **mechanism,** **outcome** **configuration** ** (CMOC)**	A description explaining the relationship between specific context(s), mechanism(s) and outcome(s). ( Duddy & Wong, 2023, p. 3).
**Initial** **programme** **theory (IPT)**	IPT/s “set out how and why a class of intervention is thought to ‘work’ to generate the outcome(s) of interest” (The RAMESES Project ( www.ramesesproject.org) ©2014 p. 4). May be expressed as “If.., then…” statements or CMOCs. It may be proven/disproven/amended during the synthesis process.
**Middle range** **programme** **theory**	The term “middle –range” is an adjective used to describe the level of abstraction of a theory. At the middle-range there is abstraction, but the theory is close enough to observable data to be usable and enable empirical testing. ( Merton, 1967; http://www.ramesesproject.org/media/RAMESES_II_Theory_in_realist_evaluation.pdf). It is expressed as CMOCs.
**Programme** **theory**	An abstracted description and/or diagram that describes what an intervention or family of interventions comprises and how it is expected to work ( Duddy & Wong, 2023).
**Demi-** **regularity**	“Semi-predictable pattern of occurrences” within data ( Cunningham *et al.,* 2021, p. 4).
**Generative** **causation**	The understanding that hidden mechanisms enact outcomes ( Jagosh, 2019).
**Retroduction**	The reasoning involved in discovering or “unearthing” causal mechanisms ( Jagosh, 2019, p. 364).

Realist review is a systematic, “theory driven, interpretative” approach ( Duddy & Wong, 2023, p. 1; Jagosh *et al.,* 2014, p. 131) that facilitates the synthesis and evaluation of data of diverse methodologies and origins. It focuses on combining empirical evidence with theoretical understanding ( Schick-Makaroff *et al.,* 2016) in programme theory development. As Wong *et al.* (2012, p. 93) state, the premise underpinning realist review is that a specific intervention or class of interventions “trigger particular mechanisms somewhat differently in different contexts”, hence, as is the case in CPT research, outcomes vary across contexts. During programme theory development context, mechanism and outcome configurations (CMOCs) are employed to coherently explain how and why contextual variables influence intervention outcomes. Ontologically rooted in realism and aligned with Bhaskar’s stratified reality ( Bhaskar, 1997), realist review explores both the visible and hidden forces that generate the outcomes of interest ( Jagosh *et al.,* 2014). From a realist standpoint, a behavioural intervention such as CPT is conceptualised to operate in open systems where the intervention changes the system and the system changes the intervention ( Pawson *et al.*, 2004; Pawson *et al.*, 2005). Realist review aims to address the real world complexity and fluidity of these operating systems by going beyond simply asking if an intervention works and exploring generative causation to determine for whom it works, under what conditions, to what extent, how and why? Employing a realist approach has the potential to enhance existing knowledge and practice in stroke specific CPT for SH&SCPs. It can build on the current evidence base, which is predominantly impairment (aphasia) and context (chronic status) specific ( Simmons-Mackie *et al.,* 2016). It can address persisting theoretical and implementation gaps by explaining the relationship between specific contextual variables and the mechanisms they trigger to enact the reasoning and responses that bring about the intended or unintended outcomes ( Dalkin *et al.,* 2015; Wong *et al.,* 2013).

Research on stroke specific CPT predominantly focuses on Aphasia ( Chang *et al.,* 2018) with an established evidence base underpinning recommendations for use of CPT for people with chronic aphasia ( Simmons-Mackie *et al.,* 2010; Simmons-Mackie *et al.,* 2016). This includes established evidence for use of CPT with medical students ( Legg *et al.,* 2005). However, given that there is a high incidence of varied and co-occurring post stroke impairments across the acute to chronic care continuum, these recommendations, while very essential in the management of aphasia, do not comprehensively address the prevailing clinical realities of health and social care professionals or SH&SCPs working with people post stroke. Chang *et al.* (2018), surveying CPT practices of 122 Australian speech pathologists found that clinicians implementing CPT in clinical stroke settings adapt aphasia specific CPT programmes for use with the broader array of impairments. Correspondingly, in the literature there is increasing recognition of the need to develop CPT for a broader range of acquired impairments that address clinical reality ( Chang *et al.,* 2018; O’Rourke *et al.,* 2018; Simmons-Mackie *et al.,* 2016; Tessier *et al.,* 2020). Studies addressing CPT for the broader range of stroke acquired communication impairments with SH&SCPs are emerging ( *e.g.,* Baylor *et al.,* 2019; Burns *et al.,* 2017; Forsgren *et al.,* 2017; Mach *et al.,* 2022). While the number of studies is small, they represent an important evolution in CPT development for SH&SCPs and this review aims to build on and potentially enhance this development. In realist review, demi-regularities or “semi-predictable patterns of occurrence” ( Cunningham *et al.,* 2021, p. 4) are explored across interventions and domains (such as health care and education) to uncover “families of mechanisms” ( Pawson, 2002, p. 344) in programme theory development. Adhering to realist philosophy, it is these “families of mechanisms” ( *e.g.,* incentivisation, persuasion) rather than “families of interventions” that trigger change and enact outcomes ( Pawson, 2002, p. 344; Wong *et al.*, 2012, p. 94). By building on current knowledge and uncovering relevant “families of mechanisms”, this review, may enhance and potentially accelerate development and implementation of stroke specific CPT for SH&SCPs in clinical education.

The need to explore an approach to data synthesis that can accommodate all valuable research data is highlighted by Simmons-Mackie *et al.* (2016). In their updated systematic review, the authors reported on the emergence of studies addressing the efficacy of CPT use with the broader range of stroke acquired communication impairments and a promising new trend of studies, attempting to manage clinical reality and the feasibility of CPT implementation in complex settings. However, all of these studies were rated too low on the American Academy of Neurology levels of evidence ( 2011) for efficacy or effectiveness to provide recommendations for clinical practice. Given that realist review accommodates the synthesis and evaluation of data of diverse methodologies and origins, it can build on existing empirical findings from systematic reviews; overcome the challenge of accommodating all valuable research findings in the final analysis, and provide clinicians and educators with augmented theoretical and practical guidance for the development and implementation of CPT.

The value of theory driven CPT interventions and implementation strategies is increasingly recognised in the literature ( Chang *et al.,* 2018; Cruice *et al.,* 2018; Shrubsole *et al.,* 2023). However, theoretical approaches are not in mainstream use and given the vast array of behaviour change and implementation theories in existence it is a challenge for researchers to select the best fit ( Eccles *et al.*, 2012; Shrubsole *et al.,* 2019). Shrubsole *et al.* (2019) argue that the potential contribution of different theories is unclear given that they have not been consistently applied to health professional’s behaviour or in this case, we argue, SH&SCPs’ behaviour. Also, as McGowan *et al.* (2020) point out, selecting only one or a few theories in behaviour change research and intervention puts the researcher at risk of omitting relevant factors. Notably, frameworks such as the Behaviour Change Wheel (BCW) ( Michie *et al.,* 2011) and the Theoretical Domains Framework (TDF) ( Atkins *et al.,* 2017; Cane *et al.,* 2012; Michie *et al.,* 2005), developed from synthesising theories and constructs, are being used more frequently in CPT research and other general research in acquired communication disorders ( Behn *et al.,* 2020; Chang *et al.,* 2018; Johnson *et al.,* 2017; van Rijssen *et al.,* 2021). A realist approach considers existing substantive theories in programme theory formation. Consequently, it has the potential to overcome “best fit” challenges while building on existing theoretical understanding. Also, unlike other theoretical approaches, which can be limited in their ability to address multiple pertinent questions, realist review applies realist logic to answer the necessary range of pertinent questions - what works, for whom, under what conditions, to what extent, how and why? ( Pawson *et al.,* 2004; Wong *et al.,* 2012; Wong *et al.,* 2013). Answering such questions can strengthen current CPT research and practice by explaining “the success, failure” and “mixed fortunes” of this complex intervention ( Wong *et al.,* 2013, p. 1). Importantly, addressing these questions allows us to look beyond determining efficacy to determining necessary solutions for real world practice in higher education institutions. The anticipated outcome of this review, a middle range programme theory (defined in Table 2) ,while abstract, will be close enough to observable data to be usable and enable empirical testing.

### Patient and Public Involvement (PPI)

PPI refers to research that is conducted ‘with’ or ‘by’ members of the public rather than ‘to’, ‘about’ or ‘for’ them ( https://www.invo.org.uk/). It involves “working in collaboration or partnership with patients, carers, service users or the public” ( https://hseresearch.ie/patient-and-public-involvement-in-research/). They may become “members of the research team, or part of reference groups, involved in key discussions and decisions, sharing their unique knowledge, expertise and perspective” ( Staniszewska *et al*., 2018, p. 1). Originating from the disability and advocacy movements, it emphasises knowledge sharing between “lay people” and “the professionals” ( McMenamin *et al*., 2022, p. 31). PPI benefits the research process, the researcher and the PPI contributors and there is increasing expectation for PPI in health and social care research ( Hersh *et al*., 2021; McMenamin *et al*., 2022). It improves quality, relevance, impact and integrity of the research process. It promotes waste avoidance and embraces active involvement including shared decision making across the research cycle ( Dawson *et al*., 2020; Hersh *et al*., 2021; McMenamin *et al*., 2022; Staniszewska & Denegri, 2013). It promotes inclusion, validation and understanding of the lived experience ( Pearl & Cruice, 2017; Turk *et al*., 2017).

### Incorporating PPI in realist review

While engagement of experts and stakeholders is commonplace in a realist review ( Saul *et al*., 2013), the inclusion of PPI is at an evolutionary stage ( Abrams *et al*., 2021). There is variability in all terms used to describe the realist review’s advisors or contributors and PPI tends to be subsumed into pre-existing structures or groups including “stakeholder, expert or advisory panels” ( Abrams *et al*., 2021, p. 243). In this review, to support clarification for the reader, we use the term PPI advisor to denote people with stroke acquired communication impairments and their significant others. They, in collaboration with content advisors, relaist advisors, student advisors and an educationalist comprise this realist review’s advisory panel (see methods, Table 3).

### Level of PPI advisors’ involvement in this realist review

**Figure 1.**
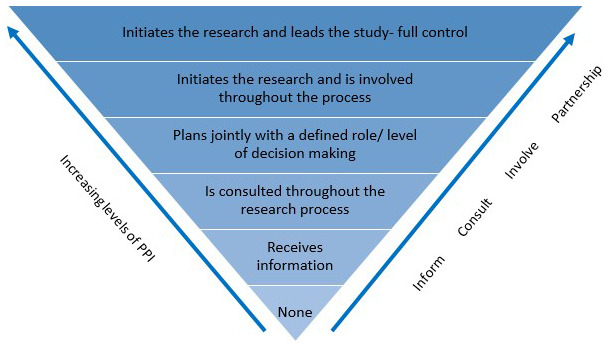
PPI Spectrum of Involvement in Research Model (IHRF, 2015 https://hrci.ie/about-us/our-work/ppi/). This figure has been reproduced with permission.

In general, PPI can occur at different levels (e.g. consultation, defining scope, collaboration) and at different stages (e.g. design, project advisor, dissemination) of a research cycle ( McMenamin *et al*., 2022). Specific to realist review Abrams *et al*. (2021) found that PPI contributor roles included informing and developing programme theory and advising over the course of the review. Developing research questions, sourcing literature and data extraction tend to be performed by advisors such as academics or clinicians. These traditional roles are reflected in the level of involvement expected from this review’s advisory panellists (see methods, Table 3). More broadly, across different types of research, there are a range of conceptual models overviewing different levels of involvement and power sharing in PPI, many of which have been influenced by Arnstein’s Ladder of Public Participation (1969) ( McMenamin *et al*., 2022). In this realist review, we adopt the PPI Spectrum of Involvement in Research Model (IHRF, 2015 https://hrci.ie/about-us/our-work/ppi/). This model, depicted in Figure 1, illustrates that the PPI advisors on this review are involved at a consultative level.

## Methods

### Aims and objectives

**Figure 2.**
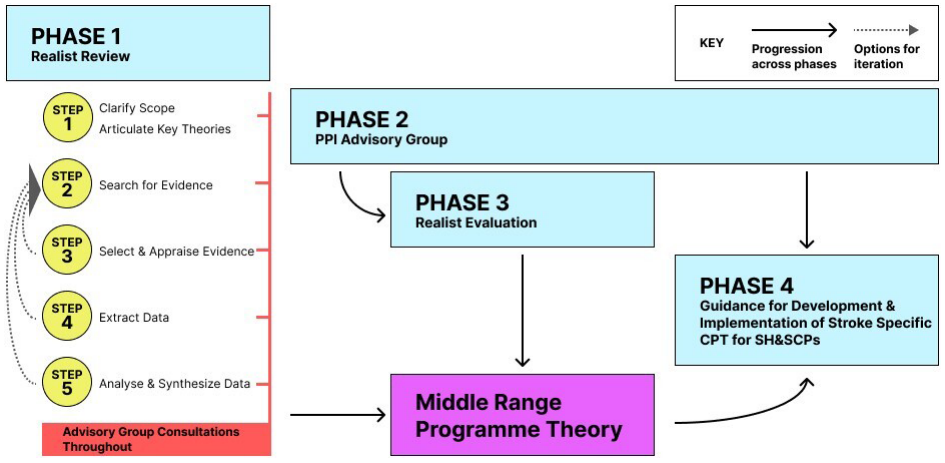
Project flow chart.

This review protocol overviews a realist review, the first phase of a PhD project. Figure 2 overviews the realist review in the context of the wider project. The wider project aims to employ a realist approach, including realist review (Phase 1) and realist evaluation (Phase 3), in combination with PPI (Phase 1- PPI advisors on review’s advisory panel and Phase 2- PPI Advisory Group). The primary aim of this realist review (Phase 1) is the synthesis of secondary data from varied relevant sources into a plausible and coherent middle range theory. This theory will explain how CPT is expected to work when teaching SH&SCPs to communicate with people with stroke acquired communication impairments. As stated above, the middle range theory developed will be close enough to observable data to be usable and permit empirical testing. It will undergo further appraisal and development during realist evaluation, the third phase of this project, in consultation with the PPI advisors who are consulting across all phases of this project. 

A protocol for this review has been registered on PROSPERO on 01/05/2023 (CRD42023418951).

### Steps in this realist review

While there are guiding principles around conducting a realist review there is no one prescribed method ( Hunter *et al.,* 2022) and this can result in variability across reviews and lack of clarity on key stages in the review process ( Booth *et al.,* 2019). This protocol aims to present a clear and transparent review process guided by Pawson *et al.*’s (2005) five key iterative steps in realist review; the Realist And Meta-narrative Evidence Syntheses—Evolving Standards quality and publication standards for realist reviews ( Wong *et al.,* 2013) and a range of protocols, methodological papers and reviews of and recommendations for practice including Booth *et al.* (2019); Dada *et al.* (2023); Duddy & Wong (2023); Rycroft-Malone *et al.* (2012); Saul *et al.* (2013), and Wong (2018). Also integral to this review is the establishment of an advisory panel to consult over the course of the review and collaboratively agree the finalised middle range programme theory. At this point, the advisory group has been established and Step 1 of the review has been completed. Step 2 is underway. Given that the review process is iterative, overlap across steps and backward forward movement is anticipated throughout the process.

### Set up review advisory panel

Duddy and Wong (2023, p. 1) describe realist review as a “flexible, iterative and practical” approach to evidence synthesis that draws on the expertise of a variety of relevant stakeholders. Consequently, when undertaking a realist review a high level and variety of knowledge and expertise is required ( Saul *et al.,* 2013). In order to realise such expertise and knowledge, this review adopted a collaborative model incorporating an advisory panel ( Davies *et al.,* 2019; Shé *et al.,* 2018). The composition of this advisory panel is overviewed in Table 3 with rational for inclusion of the specific advisors and summary of their expected roles. Also, a summary of the advisory panel meetings throughout Step 1 of the realist review is included in extended data.

**Table 3.** Realist Review Advisory Panel: Composition, inclusion rationale and overview of expected roles.

Composition	Rationale for Inclusion	Expected Roles
**Content advisors (n=4)**: Academic researchers in the field of speech and language therapy. They have specific expertise in the development and application of CPT and PPI.	Advisors with content expertise and in-depth and up to date knowledge of the subject matter are pivotal to the successful execution of the realist review ( Ní Shé *et al*., 2018; Saul *et al*., 2013).	• Directing and managing the review • Engaging in all steps of the review process • Facilitating programme theory development, review and refinement • Co-authoring academic paper • Dissemination planning and execution
**Realist advisors (n=3)**: Advisors with expertise in the realist approach.	Methodological assistance from people experienced in the realist approach is vital in the execution of the review ( Duddy & Wong, 2023).	• Methodological assistance across all steps of the review • Programme theory review and refinement • Co-authoring academic paper • •Dissemination planning and execution
**PPI advisors (n=3):** People with stroke acquired communication difficulty who are experienced co- trainers and participants on the CPT programme at University of Galway and the spouse of one of these advisors.	Involving people with the “lived experience” in research can have multiple benefits including improved quality, relevance, impact, integrity and waste avoidance ( Dawson *et al*., 2020; Hersh *et al*., 2021; Staniszewska & Denegri, 2013; Tomlinson *et al*., 2019).	• Reviewing and refining the initial programme theory (Step 1B) • Reviewing and refining the extraction template (Step 4) • Reviewing and refining the middle range programme theory (Step 5) • Dissemination planning and execution
**Student advisors (n=4):**Undergraduate students who participated on CPT at University of Galway during the academic years 2022 and 2023.	Involving students, the knowledge users, enhances the usability of the review products and strengthens links to practice ( Saul *et al*., 2013, p. 15)	•Reviewing and refining the initial programme theory (Step 1B) • Reviewing and refining the middle range programme theory (Step 5)
**Medical educationalist (n=1)** Professor in medical education.	Provides in-depth and up to date knowledge from an educational perspective.	• Reviewing and refining the initial programme theory (Step 1B) • Reviewing and refining the middle range programme theory (Step 5)

### Enabling active involvement of PPI advisors in realist review

In order to ensure the PPI advisors, especially those with communication impairments, engage actively and authentically on this advisory panel it is important to adapt a flexible and facilitative approach ( McMenamin *et al.,* 2022). Successfully negotiating complexity, such as realist theory development and refinement, with people with stroke acquired communication impairments, requires careful consideration and accommodations ( Hersh *et al.,* 2021). In this project the PhD candidate YF, is an experienced speech and language therapist who has worked with people with acquired communication difficulties for over 30 years. This according to Cascella & Aliotta (2014) renders her uniquely skilled in facilitating inclusive communicative interactions with people with acquired communication impairments. Annually, YF, RMcM and both PPI advisors with communication impairments co-deliver CPT to student speech and language therapists at the University of Galway. All are experienced in using supportive communication techniques and strategies to ensure active and reciprocal involvement during the training module. These communicative supports, tailored to the needs of each individual PPI advisor (one presenting with mild expressive aphasia and one with severe expressive aphasia and severe apraxia of speech), are being employed across this realist review to ensure understanding of the review process; clarity around the commitment and work required; active collaboration, and that there is shared understanding among the researchers and the PPI advisors. Ensuring there is comprehensive, shared understanding of the collaborative process aligns with Abrams and colleagues’ series of prompts for researchers and PPI contributors engaging in realist reviews ( https://www.spcr.nihr.ac.uk/news/blog/the-role-or-not-of-patients-and-thepublic-in-realist-reviews), and with McMenamin *et al.* (2021, p. 17) “Top Tips” for researchers including people with aphasia in research. Examples of these tips and prompts include:

• Clarifying the point of the research and whom it will benefit

• Clarifying the PPI advisors expected time and work commitments

• Clarifying language demands e.g., how much reading would be required

• Building Relationships and ensuring there is equality and trust and people with aphasia are facilitated to engage actively and authentically

• Adopting an inclusive tone and ethos

• Identifying the most appropriate communication channels for use during the review

Specific examples of supportive strategy, employed in this review for PPI advisors with communication impairments, include:

• PPI Advisor 1 – Presenting information pictorially and in short written phrases (See sample in extended data); giving extra time to process information; supplementing conversation and verbal questions and instructions with written words and symbols; facilitating the use of a variety of non-verbal modes of communication including a communication folder, smart phone and tablet.

• PPI Advisor 2 - giving extra time to process verbal and written information, presenting verbal questions and instructions “one at a time”.

Time was also dedicated to the third advisor, the spouse of contributor 1, to ensure there was shared understanding of what engaging in the review entailed. She was provided with written information and engaged in discussion around the requirements of the project. 

With regard to adopting a flexible approach, it was collaboratively agreed by YF and PPI advisors that meetings should be face to face and conducted separately with the the single advisor and the dyad (person with communication impairments and his significant other). This was due to their significantly different communication impairments and supportive requirements and to facilitate optimal use of everyone’s time.

### Step 1

A. Clarify scope

The review questions were conceptualised by the content advisors and refined in consultation with the realist advisors. The questions aim to address shortcomings in the current knowledge base and facilitate realist enquiry. In order to determine how SH&SCPs learn to communicate optimally with people with stroke acquired communication impairments, we posed the following questions:

• What CPT interventions are used for SH&SCPs in higher education institutions?

• What are the desired, achievable outcomes of CPT in higher education institutions?

• For whom do these interventions work (or not) (e.g., which SH&SCPs and at what stage of their training; do they benefit people with stroke acquired communication difficulties participating as co-trainers or communication partners)

• What contexts (e.g., organisational, pedagogical, psychosocial) are enabling/inhibitory?

• How do these interventions work (what mechanisms are enabled in specific contexts to operationalise desired/undesired/unexpected outcomes)?

B. Articulate key theories to be explored

**Figure 3.**
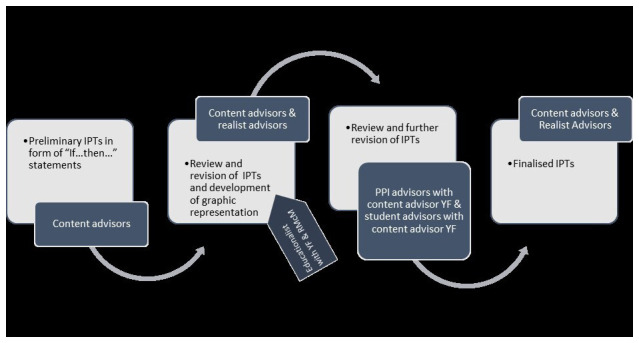
IPT development process.

Concurrent with question refinement was initial programme theory (IPT) development. This is a fundamental, early step in a realist review ( Pawson *et al.,* 2004; Wong, 2018). Our IPTs set out how CPT for SH&SCPs is thought to work. They will be subject to testing and refinement in the subsequent synthesis process of this review. Figure 3 overviews the IPTs development process.

**Figure 4.**
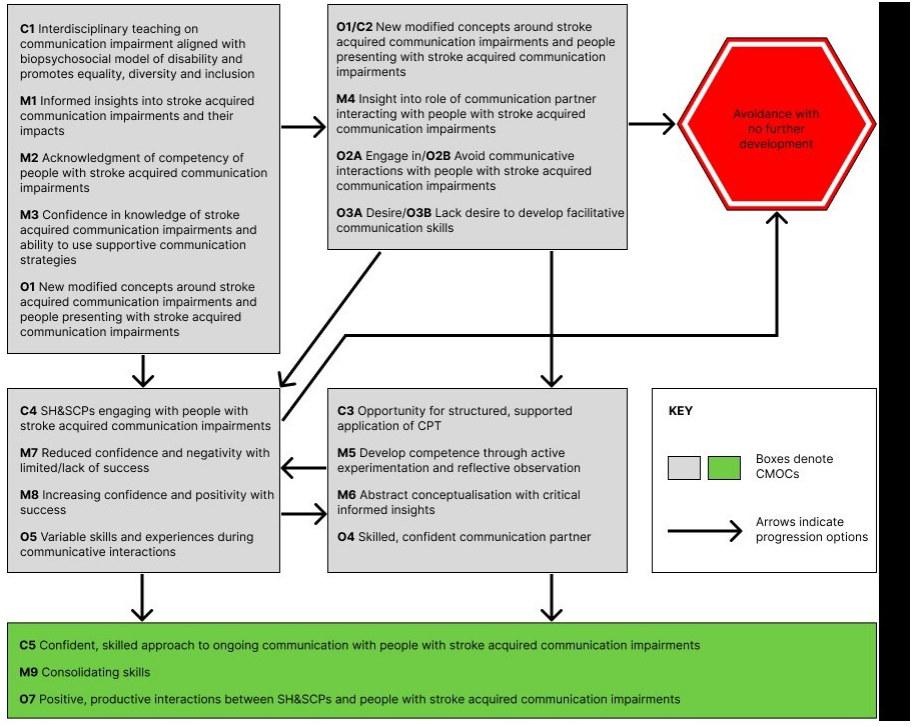
Graphic representation of integrated IPT for Stroke specific CPT for SH&SCPs.

Preliminary IPTs were developed by content advisors, guided by their insights into CPT, their knowledge of literature on the topic, and an additional literature scoping exercise (see extended data). YF formulated initial theories in the form of “If.., then…” statements. These were modified and refined iteratively over a five-month period in consultation with the other content advisors. This process generated nine “if.., then…” statements which were presented in context, mechanism, outcome configurations (CMOCs). In consultation with the realist advisors the CMOCs were developed further and amalgamated into a graphically presented, overarching IPT. This overarching graphic was additionally reviewed by a medical educationalist. Narrative and graphic IPTs were then presented to student advisors and PPI advisors for review and revision. Final revisions were agreed in consultation with content and realist advisors (see extended data). Narrative IPTs are presented in Table 4. Figure 4, graphically presents the amalgamated IPT, theorising how SH&SCPs can progress to being positive, productive communication partners, or not, and the reasoning and responses enacted along the way.

**Table 4.** Narrative presentation of IPTs for stroke specific CPT for SH&SCPs.

Context	Mechanism	Outcome
IF	THEN	AND
1. There is embedded support for the biopsychosocial model of disability in department/ school/organisation	Educators: • Are motivated to promote learning on medical and social models of disability • Recognise the value of active participation of people with stroke acquired communication difficulties in health care and society • Recognise the inherent competency of people with stroke acquired communication difficulties • Appreciate the need to develop students’ abilities to use supportive communication techniques and strategies when communicating with people with stroke acquired communication difficulties	Educators: • Desire to accommodate comprehensive CPT underpinned by social model of disability in curriculum • Given resource limitations there is a willingness to implement “condense” version of CPT, which includes perceived fundamental programme elements, into curriculum (e.g., lecture on communication impairment and supportive communication strategies and techniques).
2. There is a commitment to inter-professional learning	• There is integration of knowledge and skills • Educators appreciate the inherent value of collaborative teaching and learning opportunities	• Willingness to collaborate across disciplines to explore CPT development
3. SH&SCPs receive “condensed” CPT with perceived fundamental elements included	SH&SCPs: • Develop fundamental insights into the nature of communication disorders and generic strategies for supporting communication with people with stroke acquired communication difficulties • Feel confidence in their knowledge of communication disorders • Perceive that communicating with people with stroke acquired communication impairments will be less difficult	SH&SCPs: • Demonstrate increased willingness to communicate with people with stroke acquired communication difficulties • Anticipate success communicating with people with stroke acquired communication difficulties • Experience variable communication success with people with stroke acquired communication difficulties • Experience variable emotions, both positive (e.g., satisfaction, sense of achievement) and off putting (e.g., frustration, guilt) interacting with people with stroke acquired communication impairments • Develop some insights into effective strategy use with people with stroke acquired communication impairments • Experience increased confidence around communicating with people with stroke acquired communication impairments
4. Teaching on communication impairment prioritises the social model of disability and: • 4a. Emphasises the impact of communication on activity and participation • 4b. Promotes activity and participation of people with stroke acquired communication impairments	SH&SCPs develop: • Informed insights into the functions and importance of conversation in daily life • An appreciation of the significant impact impaired conversation skills can have on a person’s life • A desire to facilitate better conversations for people with stroke acquired communication impairments / no desire to facilitate better conversations for people with stroke acquired communication impairments due to recognition of effort required	SH&SCPs: • Will conceptualise/ reconceptualise conversation and the impact of communication impairment • Will be willing/unwilling to invest effort in developing supportive communication skills given their appreciation of the effort required • Demonstrate increased willingness to engage /avoid engaging in communicative interactions with people with stroke acquired communication impairments
	SHCPs will: • Develop critical awareness around identification of target behaviours/strategies and accommodating to the needs of people with stroke acquired communication impairments • Appreciate that communication is a shared responsibility, collaborative and co-constructed • Appreciate the need to use adaptive strategies and avoid maladaptive strategies when communicating with people with stroke acquired communication impairments • Appreciate the effort required on behalf of the communication partner to assist people with stroke acquired communication impairment reveal their competency • Recognise of the inherent competency of the people with stroke acquired communication difficulties • Realise that the communication partner has an essential role in helping reveal this competency	SHCPs: • Will be able to identify the most appropriate strategies and techniques to use with people with stroke acquired communication difficulties to optimise communication • Will be **willing** to continue to learn how to employ strategies and techniques to improve their communication with people with stroke acquired communication impairments in their daily practice and embrace communicating directly with people with stroke acquired communication impairments • Will be **unwilling** to continue to learn how to employ strategies and techniques to improve their communication with people with stroke acquired communication impairments in their daily practice (due to perceived effort and challenges) and select to communicate with significant others/staff. • View people with stroke acquired communication impairments as inherently competent
5. There is embedded support for equality, diversity and inclusion	SH&SCPs will: • Develop awareness around disability & equality; representations of disability in society; • Develop understanding of the insider experience • Desire to monitor attitudes, language and behaviours • Develop informed insights into and awareness of how disability is represented and viewed in society • Appreciate the need to adapt their attitudes/practices	SH&SCPs will: • Conceptualise/reconceptualise how disability is viewed in society • Endeavour to adapt/modify their attitudes and practices including their attitudes to and practices with people with stroke acquired communication difficulties • Demonstrate willingness to learn to become a communication partner
6a. Opportunity for structured, adequately challengingly practical application 6b. Opportunity for guided reflection on one’s own behaviour and the behaviours of others	SH&SCPs: • Develop competence through active experimentation and practice • Develop critical insights into effective use of techniques and strategies • Feel confident in their developing skill set • Believe that it is the communication partner’s responsibility to make conversation work • Develop increasingly positive attitude towards using supportive communication techniques and strategies	SH&SCPS will: • Demonstrate skilled, informed & varied use of supportive communication techniques • Have positive experiences communicating with people with stroke acquired communication difficulties • Understand how to change own behaviour to optimise communication with people with stroke acquired communication difficulties • Understand that the communication partner is vital to revealing competence of people with stroke acquired communication difficulties
	• Reflective observation of adaptive and maladaptive behaviours and communicative behaviours andattitudes • Abstract conceptualisation giving rise to new ideas/ modification of existing concepts	• New concepts/modification existing concepts (e.g., around inherent competency of people with stroke acquired communication difficulties; communication as a shared responsibility; societal view of disability; use of supportive communication)
7. Focus on rights and autonomy of people with stroke acquired communication impairments in health care practice	SH&CPs will: • Recognise the right to and value of client centre care (CCC) • Recognise the need to be competent in use of supportive communication techniques to ensure CCC for people with stroke acquired communication difficulties	SH&SCPS will: • Be willing to engage in CPT • Commit to using supportive communication for people with stroke acquired communication difficulties in facilitating CCC
8. People with stroke acquired communication impairments are engaged as co-trainers assisting in the delivery of the CPT	• There is reciprocity/mutual exchange of privileges between SH&SCPs trainers with stroke acquired communication difficulties and SH&SCPs • SH&SCPs develop insights into the lived experience • There is acknowledgement of expertise of people with stroke acquired communication difficulties • People with stroke acquired communication impairments believe in the value of training	• SH&SCP have opportunity for practice and deep learning • SH&SCPs will become empathetic, skilled communication partners • People with stroke acquired communication impairments feel valued as they are contributing to service development and enhancement • People with stroke acquired communication impairments experience positive feelings such as pride and wellbeing in helping others and improving services
9. People with stroke acquired communication difficulties participate in CPT as communication partners & / or as co-trainers	• People with stroke acquired communication difficulties experience positive social participation	People with stroke acquired communication difficulties: • Have the opportunity to meet people • Practice speaking with unfamiliar person/s • Developed increased confidence in their communication abilities • Experience decreased feelings of marginalisation

### Step 2 Search for evidence

This review aims to include a variety of articles and documents that address the review questions and can contribute to programme theory development and refinement ( Davies *et al.,* 2018; Luetsch *et al.,* 2020). In line with the realist approach this search for relevant articles and documents will be iterative and conducted in collaboration with a research services librarian. The search progression will be responsive to emergent data and developing understanding and insight ( Booth *et al.,* 2019; The RAMESES Quality Standards for Realist Synthesis, The RAMESES Project ( www.ramesesproject.org) ©2014). A concept-based search strategy, devised by YF in collaboration with the research services librarian and content experts, will direct an initial pilot search and data extraction (see Table 5). The search strategy will subsequently be refined iteratively and collaboratively with the advisory group. The searches will include peer reviewed journals, international best practice statements and clinical guidelines and conference proceedings. The following electronic data bases will be searched: Medline, EMBASE, CINAHL, APAPsycINFO and Web of Science. As outlined in our review protocol registered on PROSPERO (CRD42023418951), building from previous reviews ( Simmons-Mackie *et al.,* 2010; Simmons-Mackie *et al.,* 2016; Tessier *et al.,* 2020) searches of peer reviewed literature will be limited to the English language and by date – from January 2019 to the time of the review. Relevant studies in the previous reviews will be included for analysis. Supplementary searches will include hand searches of reference lists, and requests for unpublished studies/programmes from key authors. Search alerts will be in place to identify studies relevant for inclusion prior to final analysis.

**Table 5.** Preliminary concept-based search.

Concept 1		Concept 2		Concept 3
conversation partner training **OR** communication partner training **OR** communication strategies **OR** inclusive communication **OR** communication access **OR** supportive communication **OR** communication skills	**AND**	Aphasia **OR** Stroke **OR** Dysarthria **OR** cognitive communication disorder **OR** apraxia of speech **OR** augmentative communication **OR** assistive communication **OR** communication disorder **OR** Right hemisphere language disorder	**AND**	Nurse **OR** Doctor **OR** Medic **OR** Speech and language therapist **OR** Speech pathologist **OR** Physiotherapist **OR** Occupational therapist **OR** Psychologist **OR** Podiatrist **OR** Student **OR** health care professional **OR** Assistant **OR** patient provider **OR** Rehabilitation **OR** Allied health **OR** health care

### Step 3 Select and appraise evidence

YF will perform the Title/Abstract screening and select documents consistent with preliminary inclusion criteria, that is, CPT for health and social care professionals or SH&SCPs addressing stroke acquired communication impairments. A random 20% selection will be reviewed by RMcM. Potential disagreements will be resolved through discussion and consensus of a third author as required. Endnote 20 will be the reference management system used and Covidence 2.0 systematic review software will be used to screen titles and abstracts.

In realist reviews, inclusion of data is determined by their ability to assist in the development and refinement of programme theory or theories. Hence, multiple and varied data sources are considered for inclusion ( Wong, 2018). Applying quality scales or tools to such data sources risks excluding data that are essential for programme theory development. Therefore, in line with Price *et al.* (2021) in their realist review on remediating doctors’ performance to restore patient safety, formal quality appraisal tools will not be used in this review, on the basis that they are not sensitive to how data within papers contribute to programme theory development. Also, specific to CPT research, the application of quality scales has previously highlighted variable methodological quality of studies ( Cherney *et al.,* 2013; Simmons-Mackie *et al.,* 2016). Simmons-Mackie *et al.* (2016) recommend that the purpose of the study must also be considered when assessing its value and Pawson *et al.* (2005, p. 24) recommend that when undertaking a realist review judgement around “fitness for purpose” needs to be made.

In accordance with The RAMESES Quality Standards for Realist Synthesis The RAMESES Project ( www.ramesesproject.org) ©2014; Booth *et al.* (2013); Wong (2018), and Dada *et al.* (2023) any section of a document included in this review will be appraised for:

- Relevance
- Rigour, including plausibility, coherence, trustworthiness
- Richness

**Figure 5.**
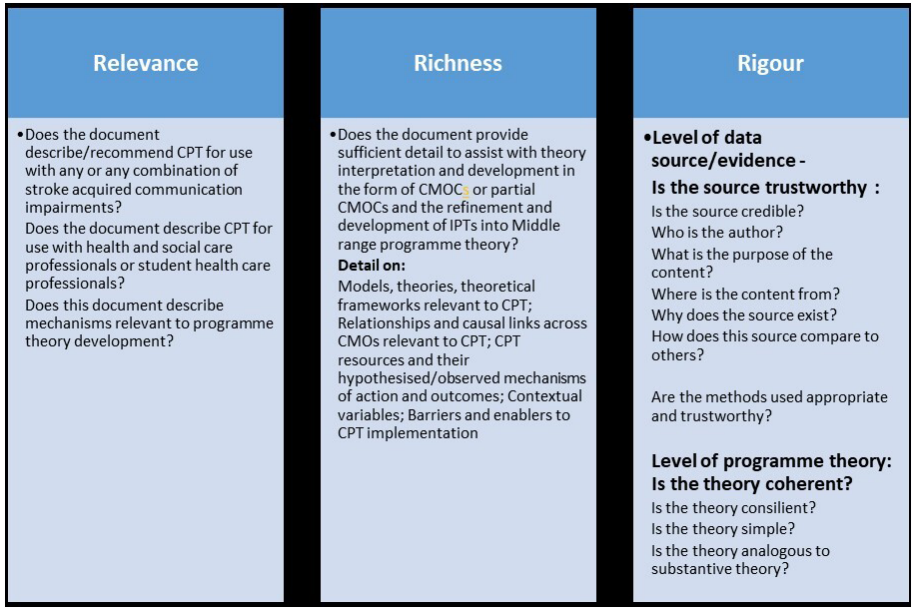
Synopsis of key concepts for appraisal of relevance, richness and rigour (adapted from Wong, 2018; Dada *et al.*, 2022 and Dada *et al.*, 2023).

Adhering to the above standards and recommendations bespoke appraisal tools are being devised for this review. Key concepts are synopsised in Figure 5.

### Step 4 Extract the data

The RAMESES Quality Standards for Realist Synthesis (The RAMESES Project ( www.ramesesproject.org) ©2014, p.7) advises that the data extraction process be continually refined over the course of the review in line with evolution of programme theory and focusing of review questions. Consequently, as Pawson *et al.* (2004, p. 23) had previously highlighted, data extraction in realist review is a process “without an exact equivalent”. A preliminary data extraction template has been devised and includes study, participant and intervention characteristics, outcome measures and outcomes, contexts, mechanisms of action and underpinning theories. For analogous purposes the domains of the substantive theoretical framework the TDF ( Atkins *et al.,* 2017) and COM-B elements of the BCW ( Michie *et al.,* 2011) are included in the extraction template. This template will be amended as indicated following the pilot searching, and iteratively thereafter in line with evolving insights and understanding. Once coded with respect to richness, relevance and rigour sufficiently rigorous data will be prioritised and assigned conceptual labels relating to CMOCs or components thereof. Less rigorous data will also be interrogated and undergo a triangulation process prior to contributing to programme theory refinement.

### Step 5 Analyse and synthesise data

Data synthesis centres on programme theory refinement ( Hunter *et al.,* 2022; Pawson *et al.,* 2005; Rycroft-Malone *et al.,* 2012). Once thoroughly familiar with the extracted data sets, interpretations and judgements will be made around:

- Partial or complete CMOC of included data
- How these data impact the refinement of the initial CMOCs developed in Step 1 and their development into middle range theory
- Whether further searching is required in response to developing insights and understanding

The following analytical process adapted from Pawson (2013) by Papoutsi *et al.* (2018, p. 16) will inform analysis and judgement throughout the synthesis process:

- Juxtaposition of data sources through comparing and contrasting data across documents
- Reconciliation of contradictory or differing data through further analysis, investigation and explanation.
- Consolidation of evidence sources where adjudications around demi-regularities can be made.

**Figure 6.**
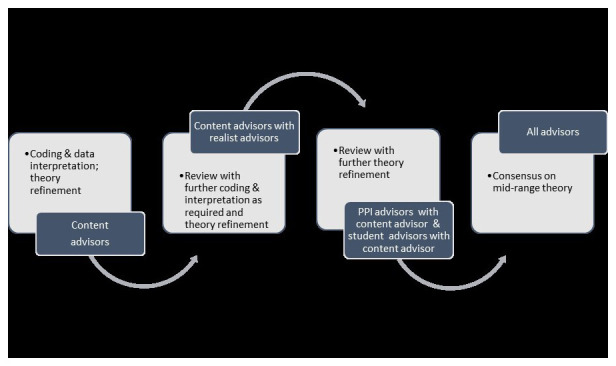
Group analysis, judgement and synthesis process.

Matrices will be devised to facilitate this analysis. Analysis, judgement and synthesis will be a collaborative and iterative group process, see Figure 6. Throughout this process uncertainties will be resolved through discussion, debate and group consensus.

### Study status

As outlined in Figure 2, this realist review comprises the first step of this project. Step one of the review has been completed and step two is underway.

### Plan for dissemination

This protocol will be submitted for publication to a peer-reviewed publishing platform. The findings of the completed review will be presented in a second article, adhering to the RAMESES publication standards for realist synthesis, and submitted for publication to a peer-reviewed journal. Also, following consultation with the advisory panel other relevant platforms including conferences, workshops, media platforms, and special interest groups will be explored for dissemination.

### Ethical approval

Ethical approval is not required for this review. No data will be collected from the advisory panel.

## Discussion and conclusion

This protocol provides an argument for using a realist approach, in conjunction with PPI, to enhance stroke specific CPT development and implementation for SH&SCPs in higher education institutions. It specifically details the first phase of this project – the realist review. People with stroke acquired communication impairments, often excluded from research that requires active participation, are consulting as members of the review’s advisory panel to improve the quality, relevance and impact of the review. They will continue to consult as PPI advisors across all phases of the project.

While research into CPT addressing the broad range of stroke acquired communication impairments is evolving, there are limited implementation guidelines and recommendations to direct practice. Aligned with this is the need to address “real world” realities and challenges in implementing complex interventions in complex clinical and educational settings. Realist review adds to existing knowledge and confronts these “real world” challenges by evaluating CPT from a theoretical and explanatory view point. In the development of a middle range programme theory it aims to coherently explain how the intervention is expected to work. While traditional systematic reviews look at what works and effect size, realist review explores generative causation in theory building and asks a much broader range of questions including who does it work for, under what conditions and how? Unlike existing systematic reviews realist review permits the synthesis of relevant and valuable data from diverse sources and methodologies. Including such varied, valuable data in the production of a middle range theory may provide educators with new and essential theoretical and practical guidance in the development and implementation of CPT programmes, tailored to their desired outcomes for a range of SH&SCPs. The middle range theory developed during this review process will undergo further analysis and development in a subsequent realist evaluation.

## Data Availability

Zenodo: Additional Supporting Data: Communication partner training for student health and social care professionals engaging with people with stroke acquired communication difficulties: A protocol for a realist review. http://dx.doi.org/10.5281/zenodo.10850179 The following supporting data is included: Materials included in scoping exercise in development of initial programme theories Sample of adapted explanatory materials for PPI advisor with severe aphasia Summary of advisory panel meetings throughout Step 1 of realist review Data are available under the terms of the Creative Commons Attribution 4.0 International license
