# Supplementary material for: Communication partner training for student health and social care professionals engaging with people with stroke acquired communication difficulties: A protocol for a realist review
Source: HRB Open Res. 2024 Apr 17;6:60. Originally published 2023 Oct 10. [Version 2] doi: 10.12688/hrbopenres.13783.2 (PMC10879762; doi:10.12688/hrbopenres.13783.2)
Supplement: Peer review history

# Reviewer report: Reviewer response for version 2

**DOI:** 10.21956/hrbopenres.15223.r39250

**Published:** 2024-06-08

**Authors:** Alexandra Tessier

**Affiliations:** 1 Centre interdisciplinaire de recherche en réadaptation et intégration sociale, Québec, Québec, Canada; 2 Orthophonie, Université du Québec a Trois-Rivières (Ringgold ID: 14847), Trois-Rivières, Québec, Canada

I finally reviewed the article again. I agree to move the status of my revision to “Approved

Is the study design appropriate for the research question?

Yes

Is the rationale for, and objectives of, the study clearly described?

Yes

Are sufficient details of the methods provided to allow replication by others?

Yes

Are the datasets clearly presented in a useable and accessible format?

Not applicable

Reviewer Expertise:

Communication partner training, communication  disabilities, environmental interventions, transportation, workplace training, participatory research

I confirm that I have read this submission and believe that I have an appropriate level of expertise to confirm that it is of an acceptable scientific standard.

# Reviewer report: Reviewer response for version 2

**DOI:** 10.21956/hrbopenres.15223.r39247

**Published:** 2024-05-24

**Authors:** Marina Charalambous

**Affiliations:** 1 CO- REsearch Lab (COREL), Department of Rehabilitation Sciences, School of Health Sciences, Cyprus University of Technology, Limassol, Cyprus

Thank you for thoroughly addressing all my comments, and I wish you the best of luck in the next phases of your study.

Is the study design appropriate for the research question?

Yes

Is the rationale for, and objectives of, the study clearly described?

Yes

Are sufficient details of the methods provided to allow replication by others?

Partly

Are the datasets clearly presented in a useable and accessible format?

Yes

Reviewer Expertise:

Patient and Public Involvement (PPI) in stroke and aphasia research

I confirm that I have read this submission and believe that I have an appropriate level of expertise to confirm that it is of an acceptable scientific standard.

# Reviewer report: Reviewer response for version 1

**DOI:** 10.21956/hrbopenres.15077.r37741

**Published:** 2024-03-04

**Authors:** Alexandra Tessier

**Affiliations:** 1 Centre interdisciplinaire de recherche en réadaptation et intégration sociale, Québec, Québec, Canada; 2 Orthophonie, Université du Québec a Trois-Rivières (Ringgold ID: 14847), Trois-Rivières, Québec, Canada

First of all, thank you for the opportunity to review this protocol, which describes an ambitious and impressive project. Specifically, this protocol describes how the authors, using a realist review methodology, will develop a middle-range theory to explain the functional mechanisms of CPT offered to student health and care professionals in an academic context. This project addresses the needs of settings for the real implementation of CPT and aims to fill gaps in the scientific literature on CPT offered to student health and care professionals. It seems important for realizing an inclusive society, providing quality care, and advancing scientific knowledge on CPT to explain how to implement this intervention in real life. The presence of an advisory board, including individuals with experiential knowledge, is a notable strength of the study that contributes to ensuring its rigor and relevance for all. I look forward to reading the results of this realist review.

**Introduction**

 The rationale justifies clearly and convincingly the relevance of conducting the proposed research project. The literature review is exhaustive and is supported by relevant sources in the field. I particularly appreciated that the authors explained the realist review methodology in sufficient detail in the introduction, as it is a methodology that I was not familiar with. This study addresses clinical and scientific needs to identify realistic ways to implement CPT in different settings (here for student health and social care professionals). I noticed a few typos that could be corrected:

- Table 1 → Aphasia → spelling mistake "it may be difficult to verbalize" and not "difficulty".
- Introduction, third paragraph, please introduce abbreviation Sh&SCP after the first time you fully write it (line 3 of the 3rd paragraph)

**Methods**

 The study design is appropriate for the research question and the methodology is described in detail. I particularly appreciated the numerous methodological references that the authors draw upon and the figures summarizing the processes and results that the team has obtained so far. However, I must mention again that I am not familiar with the realist review methodology. Therefore, I am not able to judge if important points in explaining the methodology are missing. Nevertheless, in my opinion, the methodology is sufficiently detailed and referenced to allow replication based on the protocol and the studies cited.

  I have some questions and suggestions for clarification for the methodology section of the protocol:

- Research question 4 (For whom…), in the examples, when you say "people with communication difficulties", what do you mean? Like, with what types of communication difficulties do CPT have effects? Or what type(s) of communication difficulties are addressed in CPT? It's not quite clear to me what this example evokes here.
- In the paragraph where supported conversation is mentioned to support the participation of people with communication disability on the advisory board: what are the "top tips" that you adhere to (McMenamin et al., 2021)? Since I am not familiar with the article in question, I would have appreciated a summary of what it specifically entails.
- I wonder if the research questions presented in the second part of the method should rather be presented under "step 1". That way, we would know upfront who developed the questions and their objectives before reading them. I don't think it would impair my understanding of the method if it were moved down, and it would thus follow the chronological order of the process in the presentation, which I believe would aid in the general understanding of the methodology.
- Table 4. In concept 2, could it be relevant to add to the search strategy "communication disability*" and "communication impairment*"?

Is the study design appropriate for the research question?

Yes

Is the rationale for, and objectives of, the study clearly described?

Yes

Are sufficient details of the methods provided to allow replication by others?

Yes

Are the datasets clearly presented in a useable and accessible format?

Not applicable

Reviewer Expertise:

Communication partner training, communication  disabilities, environmental interventions, transportation, workplace training, participatory research

I confirm that I have read this submission and believe that I have an appropriate level of expertise to confirm that it is of an acceptable scientific standard, however I have significant reservations, as outlined above.

# Response

**Published:** 2024-03-21

**Authors:** Yvonne Fitzmaurice

**Affiliations:** School of Health Sciences, University of Galway, Galway, County Galway, Ireland

Dr Tessier, thank you for taking the time to review out protocol. We very much appreciate your encouraging comments, the questions you have posed and the recommendations you have made. We have made some amendments to our protocol, in response to your and three other reviewers’ recommendations. Additionally, we have addressed all of your suggestions and queries below.

**Introduction**

**I noticed a few typos that could be corrected: Table 1 → Aphasia → spelling mistake "it may be difficult to verbalize" and not "difficulty".**

 Thank you, this typo has been corrected.

**Introduction, third paragraph, please introduce abbreviation Sh&SCP after the first time you fully write it (line 3 of the 3rd paragraph)**

 Thank you, we have included this amendment.

**Methods**

**I have some questions and suggestions for clarification for the methodology section of the protocol: Research question 4 (For whom…), in the examples, when you say "people with communication difficulties", what do you mean? Like, with what types of communication difficulties do CPT have effects? Or what type(s) of communication difficulties are addressed in CPT? It's not quite clear to me what this example evokes here.**

 Thank you for this question. We mean people with stroke acquired communication difficulties participating as co-trainers or communication partners. We want to explore if CPT works for them (and in our final question we want to explore how it works for them). For example, are the outcomes hypothesised in CMOC 9 realized? Do they develop increased confidence in their communication abilities; do they have the opportunity to meet people; do they practice speaking with unfamiliar people; do they experience decreased feelings of marginalisation?

 We have amended our question in response to your query to increase clarity.

**In the paragraph where supported conversation is mentioned to support the participation of people with communication disability on the advisory board: what are the "top tips" that you adhere to (McMenamin et al., 2021)? Since I am not familiar with the article in question, I would have appreciated a summary of what it specifically entails.**

 Thank you for this suggestion. Example of what the tips entail have been added.

 I **wonder if the research questions presented in the second part of the method should rather be presented under "step 1". That way, we would know upfront who developed the questions and their objectives before reading them. I don't think it would impair my understanding of the method if it were moved down, and it would thus follow the chronological order of the process in the presentation, which I believe would aid in the general understanding of the methodology.**

 Questions have been moved to Step 1.

Table 4. In concept 2, could it be relevant to add to the search strategy "communication disability*" and "communication impairment*"

 Thank you for your suggestion. When developing our search strategy, we were assisted by a specialist librarian and trialled several terms and combinations of terms before settling on our final concepts. We are happy that the search has returned very relevant articles and are currently at the data extraction and analysis and synthesis steps of the review.

# Reviewer report: Reviewer response for version 1

**DOI:** 10.21956/hrbopenres.15077.r37743

**Published:** 2024-02-20

**Authors:** Rachael Rietdijk

**Affiliations:** 1 The University of Sydney, Sydney, New South Wales, Australia

The authors have outlined a comprehensive plan for conducting a realist review on the important topic of communication partner training for student health and social care professionals. The paper integrates references to a large body of relevant literature to support the development of a compelling case for the review. I offer the following points of feedback which may assist to improve the clarity of the paper:

 Title and abstract: Consider including the word "protocol" in the title and when describing the methods. For example, the title could state, "A protocol for a realist review". This change would help to set the reader's expectations for the content of the paper.

 Introduction: The introduction provides a clear overview of communication impairments after stroke and communication partner training. The introduction to the approach of realist review is reasonably clear to me as a reader who is not familiar with this area. Could the authors support their explanation with an illustrative example of how this approach has been used in a similar field? A definition of the term "middle range theory" would also be useful to add to the text in this section. I would also suggest moving Table 2 to be referenced in this section of the introduction, as these definitions will be helpful to readers unfamiliar with this topic.

 Methods: The research questions are clearly articulated, and methods are well described and rationalised. I noted the following queries regarding this section:

 - Aims and objectives: Figure 1 clearly outlines the phases and steps of the project, but this could be described more clearly in the text. For example, the first phase and third phase of the project are defined in the text, but the second and fourth phases are not mentioned. The reference to Figure 1 could also be moved to this section.

 - Questions: Could you provide some examples of "contexts" (in the same way that you have given some examples of what is meant by "whom" in the previous question.)

 - Review advisory group: I was interested in a little more information on how the advisory group works (e.g., How often does the group meet? In-person or online? Are all members present at all meetings, or are there smaller sub-groups which meet to discuss specific issues? Are there any reimbursement arrangements in place?). I was also interested to know if all the student advisors all from an SLT program, or are they from other backgrounds?

 Step 1: Table 3 is well presented and illustrates the IPT concepts well. I had more difficulty following Figure 3. Could you share more about the process that lead to grouping the C/M/O elements in this way? Do learners always start in the top left hand box, or can they start in any box? 

 Step 2: The search terms appear to focus on student health care professionals, rather than social care professionals. Were other search terms considered (e.g., social work, psychology, counselling, etc?)

 Discussion: The authors conclude with a reflection on the benefits of a realist approach for addressing the research questions. The primary point mentioned here is the benefit for educators. I would be interested to read more on the authors' thoughts about the potential benefits of this project for students and for people with communication impairments.

 Minor typographical errors:

 In the introduction, "real word complexity" should be "real world complexity"

 In the final sentence, "subsequent"

 I wish the authors success with this important project and hope that this feedback is helpful as you continue your work in this area.

Is the study design appropriate for the research question?

Yes

Is the rationale for, and objectives of, the study clearly described?

Yes

Are sufficient details of the methods provided to allow replication by others?

Partly

Are the datasets clearly presented in a useable and accessible format?

Yes

Reviewer Expertise:

Communication partner training after acquired brain injury

I confirm that I have read this submission and believe that I have an appropriate level of expertise to confirm that it is of an acceptable scientific standard.

# Response

**Published:** 2024-03-21

**Authors:** Yvonne Fitzmaurice

**Affiliations:** School of Health Sciences, University of Galway, Galway, County Galway, Ireland

Dear Dr Rietdijk, thank you for taking the time to review this protocol. We appreciate your very helpful advice. We have made some amendments to our protocol, in response to your and three other reviewers’ recommendations. Additionally, we have addressed all of your suggestions and queries below.

**Title and abstract**

**Consider including the word "protocol" in the title and when describing the methods. For example, the title could state, "A protocol for a realist review". This change would help to set the reader's expectations for the content of the paper.**

 Thank you, the authors have taken on board your suggestion and amended the title.

**Introduction**

**The introduction to the approach of realist review is reasonably clear to me as a reader who is not familiar with this area. Could the authors support their explanation with an illustrative example of how this approach has been used in a similar field?**

 Thank you for this suggestion. While an illustrative example of how this approach has been used in a similar field has not been incorporated in this review protocol, the authors agree that this would be a very helpful addition and will explore including such an example when reporting on the completed review.

**A definition of the term "middle range theory" would also be useful to add to the text in this section.**

 Thank you, reference to "middle range theory" this has been added to the introduction under Employing realist review in CPT research, end of paragraph 4.

**I would also suggest moving Table 2 to be referenced in this section of the introduction, as these definitions will be helpful to readers unfamiliar with this topic.**

 We have requested that the editors move this table.

**Aims and Objectives**

**Figure 1 clearly outlines the phases and steps of the project, but this could be described more clearly in the text. For example, the first phase and third phase of the project are defined in the text, but the second and fourth phases are not mentioned.**

**The reference to Figure 1 could also be moved to this section.**

 Thank you, reference to the second and fourth phases has now been included.

 The reference to Figure 1 has been moved to this section.

**Questions**

**Could you provide some examples of "contexts" (in the same way that you have given some examples of what is meant by "whom" in the previous question.)**

 Thank you, we have included examples.

**Review advisory panel**

**I was interested in a little more information on how the advisory group works (e.g., How often does the group meet? In-person or online? Are all members present at all meetings, or are there smaller sub-groups which meet to discuss specific issues? Are there any reimbursement arrangements in place?). I was also interested to know if all the student advisors all from an SLT program, or are they from other backgrounds?**

 Subgroups met (always with one or all content experts) as direct by the content experts during initial programme theory development. Additional information has been added on the review advisory panel in Table 3 (e.g., origins of student advisors) and in the newly added extended data (Summary of advisory panel meetings throughout Step 1 of realist review)

 There were no reimbursement arrangements in place at the outset. This is not a funded PhD.

**Step 1**

**Table 3 is well presented and illustrates the IPT concepts well. I had more difficulty following Figure 3. Could you share more about the process that lead to grouping the C/M/O elements in this way? Do learners always start in the top left hand box, or can they start in any box?**

 Figure 3 aims to provide a graphic summary of the nine CMOCs detailed in Table 3 and depict that “outcome routes” can be variable and follow planned or unplanned trajectories. Also, it depicts that outcomes can be reached at different rates and be both desirable and undesirable.

 The content advisors grouped the original nine CMOCs into the boxes and arrows graphic and refinements were made in consultation with all other advisors. Additional details of meetings conducted during this process have been added in the extended data.

 It is not necessary for the reader to start in the top left hand box.

**Step 2**

**The search terms appear to focus on student health care professionals, rather than social care professionals. Were other search terms considered (e.g., social work, psychology, counselling, etc?)**

 Yes, other search terms including psychology were included.

**Discussion**

**The authors conclude with a reflection on the benefits of a realist approach for addressing the research questions. The primary point mentioned here is the benefit for educators. I would be interested to read more on the authors' thoughts about the potential benefits of this project for students and for people with communication impairments**

 This review has progressed to the extraction and synthesis phases and potential benefits to all relevant parties are becoming increasingly clear. We aim to report further about the potential impacts of this project for students and for people with communication impairments on the completion of the review.

**Typographical errors**

**In the introduction, "real word complexity" should be "real world complexity" In the final sentence, "subsequent"**

 Thank you, these errors have been rectified.

# Reviewer report: Reviewer response for version 1

**DOI:** 10.21956/hrbopenres.15077.r37731

**Published:** 2024-02-13

**Authors:** Ariné Kuyler

**Affiliations:** 1 University of Pretoria, Pretoria, Gauteng, South Africa

Thank you for the opportunity to review this manuscript. Although the manuscript has merit various questions arose.

**Introduction**

- Please very clearly define who you refer to when using the terms student health and social care professionals, as individuals from different countries or backgrounds can interpret this differently.
- You mention that student health and social care professionals engage with people with stroke-acquired communication disorders in tertiary education. However, it is unclear in what context these individuals are accessed.  Do the individuals with stroke receive therapy or some form of social support or community engagement at the institution??? Or are you referring to student undergraduate education on CPT for persons with stroke?
- Additionally, are the student health and social care professionals acting as communication partners? Are they familiar or unfamiliar to the person and also what care pathway is taken for persons with stroke-acquired communication disorders to end up with these professionals?
- Also, you interchangeably refer to student health and social care professionals and healthcare providers, please use one term consistently throughout your manuscript.
- You mention that this review may enhance and potentially accelerate development and implementation specific to CPT related to stroke in clinical education. How will the review accomplish this?
- All healthcare providers worldwide are required to provide ethical and evidence-based practice. I can understand that a realist view can be of benefit but how does a realist view correlate with actual evidence-based practice? To provide ethical services a combination needs to be used of theory, clinicians' experience and the reported needs of individuals with stroke and their family members. I am unsure how you are incorporating these aspects.

**Questions:**

- Are you looking at the actual implementation of CPT in higher education settings or what the students for example in speech-language pathology are being taught during their undergraduate degree???
- Please specify the context as your questions are too broad and unclear.
- Rephrase the question: What CPT interventions are used for SH & SCPs in higher education institutions? To read as follows: What CPT interventions are included in undergraduate curriculums for SH and SCPs at higher education institutions?
- Rephrase for whom the interventions work or not. A suggestion would be to rather say, what are the beneficial outcomes reported by participants of CPT (including both the person and communication partner)?
- Desired outcomes and achievable outcomes are very different things. You can change the question to read as follows: How are outcomes measured in CPT interventions taught to students in undergraduate curriculums at higher education institutions?
- Rephrase What contexts are enabling/inhibitory? To read as What contexts act as facilitators or barriers for the successful implementation of CPT?
- How these interventions work is a very broad question. Are you referring to the characteristics of the programme??
- Additionally, are you also thinking of how knowledge and skills taught in CPT are maintained after the intervention is completed?

Is the study design appropriate for the research question?

Yes

Is the rationale for, and objectives of, the study clearly described?

Partly

Are sufficient details of the methods provided to allow replication by others?

Yes

Are the datasets clearly presented in a useable and accessible format?

Yes

Reviewer Expertise:

Communication-partner training; adults with acquired neurogenic communication disorders; palliative care; ICU care; family-centred care

I confirm that I have read this submission and believe that I have an appropriate level of expertise to confirm that it is of an acceptable scientific standard, however I have significant reservations, as outlined above.

# Response

**Published:** 2024-03-21

**Authors:** Yvonne Fitzmaurice

**Affiliations:** School of Health Sciences, University of Galway, Galway, County Galway, Ireland

Dr Kuyler, thank you for taking the time to review our realist review protocol. We are very grateful for your recommendations and questions. We have made some amendments to our protocol, in response to your and three other reviewer’s recommendations. Additionally, we have addressed all of your recommendations and queries below.

**Introduction**

**Please very clearly define who you refer to when using the terms student health and social care professionals, as individuals from different countries or backgrounds can interpret this differently**.We have added additional detail in our introduction (paragraph 3, lines 2 & 3).

 We have included *“pre-registration”* in the amended statement below to further clarify what we mean by the word “student”.

**You mention that student health and social care professionals engage with people with stroke-acquired communication disorders in tertiary education. However, it is unclear in what context these individuals are accessed.  Do the individuals with stroke receive therapy or some form of social support or community engagement at the institution? Or are you referring to student undergraduate education on CPT for persons with stroke?**

 When referring to student health and social care professionals engaging with people with stroke acquired communication difficulties, we purposely adopted a general contextual stance to accommodate the range of students and the range of activities that they could potentially engage in across disciplines and institutions. We did not intend to specify one specific activity, set of students or institution.

 We have included the following amendment to the introduction to increase clarity:

 “Student health and social care professionals (SH&SCPs) *who may* *also, in contexts where professional registration is required, be referred to as  pre-registration professionals*, experience similar emotions when interacting with people with stroke acquired communication impairments. *Over the course of their clinical training such students may be exposed to a variety of pedagogies and engage in a range of clinical activities and placements involving people with stroke acquired communication impairments.*****

**Additionally, are the student health and social care professionals acting as communication partners? Are they familiar or unfamiliar to the person and also what care pathway is taken for persons with stroke-acquired communication disorders to end up with these professionals?**

 The student health and social care professionals referred to in the introduction are not necessarily acting as communication partners.

 Later, as we search for and appraise the evidence in Steps 2 and 3 of the review, we only include documents reporting on students engaging in CPT.  These fall under the umbrella of CPT for unfamiliar communication partners. People with stroke acquired communication impairments do not participate on all of these training programmes. Data extraction is currently in train and our findings will be reported on in a future publication, following completion of the review process.

**Also, you interchangeably refer to student health and social care professionals and healthcare providers, please use one term consistently throughout your manuscript.**

 “Healthcare providers” was used once when citing Manning et al., 2019. It was not used interchangeably with SH&SCPs and was referring to what Manning and colleagues labelled healthcare professionals. We have inserted the term used by these authors for increased clarity. “Furthermore, people with aphasia believe that training **healthcare professionals** the use of communication strategies is important in enabling them to live successfully with the communication impairment (Manning et al., 2019)”

**You mention that this review may enhance and potentially accelerate development and implementation specific to CPT related to stroke in clinical education. How will the review accomplish this?**

 At the beginning of this sentence, we state that the review may accomplish this “By building on current knowledge and uncovering relevant “families of mechanisms”.  “Families of mechanisms” is explained in greater detail in the preceding paragraph. Additionally, in our discussion and conclusion we provide further support for this argument.

**All healthcare providers worldwide are required to provide ethical and evidence-based practice. I can understand that a realist view can be of benefit but how does a realist view correlate with actual evidence-based practice? To provide ethical services a combination needs to be used of theory, clinicians' experience and the reported needs of individuals with stroke and their family members. I am unsure how you are incorporating these aspects.**

 A realist review adds theoretical and explanatory evidence to the evidence base. Unique to systematic data synthesis approaches, it incorporates an advisory panel (in our review comprising of PPI advisors with lived experience of stroke, student advisors who have participated on CPT, content advisors who have implemented CPT, realist advisors and an educational advisor) that consults throughout the review process.  In our review this panel will collectively agree the finalised programme theory. The outcome of this review will be a middle range programme theory explaining how CPT is expected to be operationalised by educators in higher education institutions and our advisory panel reflects the particular needs of this demographic. Clinical, educational, emic and end user expertise is harnessed in theory development.

 This middle range programme theory will be developed further during the subsequent phases of this research project (See Figure 1) and, in due course, reported on in further publications. The ultimate aim (Phase 4) is to provide guidance for educators wishing to implement CPT for their students. The focus on contextual variables during theory development will enhance implementation efforts for individual educators in variable contexts. However, actual implementation- the next step, is beyond the scope of this project and will require further attention to the issues you raise, specific to the educational setting.

**Questions**

**Are you looking at the actual implementation of CPT in higher education settings or what the students for example in speech-language pathology are being taught during their undergraduate degree?**

 No, as outlined in the response above, this is not looking at  actual implementation and, it is not considering what is being taught on the SLT curriculum

**Please specify the context as your questions are too broad and unclear.**

 Context is defined in Table 2 as “Any condition that triggers and/or modifies the behaviour of a mechanism” in the generation of outcomes (Duddy & Wong, 2023 p.3). Contexts may be social, psychological, material, organisational, economic etc. (http://www. ramesesproject.org/media/RAMESES_II_Context.pdf).

 We have amended one question to add examples of context - What contexts *(e.g., organisational, pedagogical, psychosocial)* are enabling/inhibitory?

 Three other reviewers do not concur with this statement that questions are too broad and unclear. One reviewer has stated that all questions have been “clearly articulated”.

**Rephrase the question: What CPT interventions are used for SH & SCPs in higher education institutions? To read as follows: What CPT interventions are included in undergraduate curriculums for SH and SCPs at higher education institutions?**

 We have not changed this question in line with the response above. Also, not all students are at undergraduate level.

**Rephrase for whom the interventions work or not. A suggestion would be to rather say, what are the beneficial outcomes reported by participants of CPT (including both the person and communication partner)?**

 Thank you for your suggestion. This question was designed to align with realist inquiry. “Realist review applies realist logic to answer the necessary range of pertinent questions - what works, for whom, under what conditions, to what extent, how and why? (Pawson et al., 2004; Wong et al., 2012; Wong et al., 2013). The structure of the question will allow for exploration of  CPT  “successes” and “failures” (Wong et al, 2013 p. 1)

 We have amended the question as follows: “For whom do these interventions work (or not) (e.g., which SH&SCPs and at what stage of their training; *do they benefit people with* *stroke acquired communication difficulties participating as co-trainers or communication partners)?* *”*

**Desired outcomes and achievable outcomes are very different things. You can change the question to read as follows: How are outcomes measured in CPT interventions taught to students in undergraduate curriculums at higher education institutions?**

 Thank you for your suggestion. However, determining *how outcomes are measured* is not a primary focus of this review. In this protocol we are interested in *exploring how CPT works* to bring about the outcomes that are both desirable and achievable in the specific contexts that they are delivered.

**Rephrase What contexts are enabling/inhibitory? To read as What contexts act as facilitators or barriers for the successful implementation of CPT?**

 Thank you for your suggestion. Prior to articulating this question, we debated the use of terms including the terms facilitators and barriers. We argue that the terms enablers and facilitators and barriers and inhibitors are used interchangeable in the literature, and prefer to retain the original text in the question. As stated above we have added examples of context to this question.

**How these interventions work is a very broad question. Are you referring to the characteristics of the programme?**

 No, we are not referring to the characteristic of a single programme, rather we are referring to CPT programmes in general and the mechanisms that are triggered (i.e., reasonings and responses) in specific contexts to explain potential outcomes. The question “How do these interventions work” explores what mechanisms are enabled in specific contexts to operationalise desired/undesired/ unexpected outcomes and is integral to realist enquiry and programme theory development.

**Additionally, are you also thinking of how knowledge and skills taught in CPT are maintained after the intervention is completed?**

 Yes, we are, in our exploration of how CPT works.  Of note, to date in our data extraction process, just one study has addressed this query (Power et al., 2023). Our findings will be reported on in detail in due course.

# Reviewer report: Reviewer response for version 1

**DOI:** 10.21956/hrbopenres.15077.r36931

**Published:** 2023-11-21

**Authors:** Marina Charalambous

**Affiliations:** 1 CO- REsearch Lab (COREL), Department of Rehabilitation Sciences, School of Health Sciences, Cyprus University of Technology, Limassol, Cyprus

Dear Authors,

 Thank you for the opportunity to review your study protocol on "Communication partner training for student health and social care professionals engaging with people with stroke acquired communication difficulties: A realist review." Your study protocol explores the complex landscape of stroke-specific communication partner training using a realist review approach, which I find suitable for the aim of the project.

 The authors effectively contextualize in the introduction the importance of communication rehabilitation for individuals who have had a stroke. They also emphasize the crucial role of communication partner training, particularly for student health and social care professionals. The review's methodology is considered appropriate. Also, the authors suggest using a Patient and Public Involvement (PPI) approach throughout the research process, which is suitable for the project's scope. However, the introduction lacks important information on PPI. To make the manuscript more informative for the reader on PPI, I suggest adding the following to the introduction:

1.  The definition of PPI (National Institute of Health Research, 2014 or other relevant sources),
2.  A brief description of the different types/levels of PPI (Arnstein’s work on the ladder of participation in 1969),
3.  An explanation of why the formation of the ‘advisory groups’ was selected compared to active PPI partnership (Charalambous et al., 2022; Mc Menamin et al., 2022).

Additionally, it would be helpful to add a paragraph in the methods section specifically explaining the following:

1. How were the advisory groups created? It seems that the advisory groups do not participate equally in the project.
2. Did other advisors besides the content and realist advisors play any role in formulating the research questions and deciding on the design of the study?
3. What were the participatory methods employed in the project?
4. What is the PPI framework that the authors will use to design and monitor the contributions of the 'people with lived experience of stroke acquired communication impairments/PPI advisors'? Referring to the PAOLI framework developed by Charalambous and colleagues in 2023.
5. How will the authors approach the PPI patients in all advisory groups to avoid tokenism, especially those with communication difficulties?
6. What are the obligations of the 'People with lived experience of stroke acquired communication impairments/PPI advisors' in the research team?
7. What are the contributions expected from all PPI advisory groups in each phase of the project? Please briefly explain how involving PPI will improve the study's impact and the implementation of the results.

This information will ensure transparent documentation of PPI and especially patient advisors’ contributions. It would be useful to include a table showing expected/completed PPI contributions from each advisor group in each project phase.

 It would be helpful to provide additional demographic data on the two PPI advisors who have lived experience of stroke and related communication impairments. This data could include information such as the type and severity of the communication impairment, time since the stroke, previous and current employment, marital status, level of education, and psychosocial information. This information would provide the reader with a clearer understanding of the patient advisors' level of involvement within the research team.

 While the review is yet to reveal its findings, the anticipation is high for insights that extend beyond mere program efficacy. By exploring the 'why' and 'how' behind communication partner training effectiveness, the study has the potential to provide actionable recommendations for refining and tailoring interventions to the unique needs of individuals with stroke-induced communication impairments. To ensure that the findings of the study are robust and applicable across diverse contexts, it's crucial to acknowledge and address several factors such as the diversity in stroke manifestations, varying communication needs, and the dynamic nature of healthcare settings.

 It is suggested that the authors provide a more comprehensive explanation of their approach to supporting PPI patient advisors throughout phase 2 of the project, with particular attention to addressing their communication challenges. This information will be useful for other researchers who wish to learn how to better support team members with communication difficulties. Finally, including patients and the public in research communication and dissemination strategies helps bridge the gap between scientific findings and the broader community. Please describe how the two PPI advisors who have lived experience of stroke and related communication impairments will be involved in disseminating the project’s outcomes.

 I hope that the suggestions I have provided will be helpful. Good luck with your project.

Is the study design appropriate for the research question?

Yes

Is the rationale for, and objectives of, the study clearly described?

Yes

Are sufficient details of the methods provided to allow replication by others?

Partly

Are the datasets clearly presented in a useable and accessible format?

Yes

Reviewer Expertise:

Patient and Public Involvement (PPI) in stroke and aphasia research

I confirm that I have read this submission and believe that I have an appropriate level of expertise to confirm that it is of an acceptable scientific standard, however I have significant reservations, as outlined above.

## References

[1] : Patient and public involvement in health and social care research: a handbook for researchers by research design service London. *London: NIHR* .2014;

[2] : A Ladder Of Citizen Participation. *Journal of the American Institute of Planners* .1969;35(4) : 10.1080/01944366908977225 216-224 10.1080/01944366908977225

[3] : The views of people living with chronic stroke and aphasia on their potential involvement as research partners: a thematic analysis. *Research Involvement and Engagement* .2022;8(1) : 10.1186/s40900-022-00379-1 10.1186/s40900-022-00379-1 36064623 PMC9446531

[4] : Distinctions and blurred boundaries between qualitative approaches and public and patient involvement (PPI) in research. *International Journal of Speech-Language Pathology* .2022;24(5) : 10.1080/17549507.2022.2075465 515-526 10.1080/17549507.2022.2075465 35762365

[5] : The development of the People with Aphasia and Other Layperson Involvement (PAOLI) framework for guiding patient and public involvement (PPI) in aphasia research. *Research Involvement and Engagement* .2023;9(1) : 10.1186/s40900-023-00484-9 10.1186/s40900-023-00484-9 37658465 PMC10474738

# Response

**Published:** 2024-03-21

**Authors:** Yvonne Fitzmaurice

**Affiliations:** School of Health Sciences, University of Galway, Galway, County Galway, Ireland

Dr Charalambous, thank you for taking the time to review our protocol. We are very grateful for questions you have posed and the recommendations you have made. We have made some amendments to our protocol, in response to your and three other reviewers’ recommendations. Additionally, we have responded to all your queries and recommendations below.

**To make the manuscript more informative for the reader on PPI, I suggest adding the following to the introduction:**

**The definition of PPI (National Institute of Health Research, 2014 or other relevant sources)**

**A brief description of the different types/levels of PPI (Arnstein’s work on the ladder of participation in 1969)**

**An explanation of why the formation of the ‘advisory groups’ was selected compared to active PPI partnership (Charalambous et al., 2022; Mc Menamin et al., 2022).**

 Thank you for the recommendation for the introduction section. The following edits have been made:

 The INVOLVE definition adopted for the National Institute for Health Research (NIHR) of PPI has been included in a new PPI section added to the introduction.

 Arnstein’s Lader of Participation and the PPI Spectrum of Involvement in Research Model (IHRF, 2015) have been referenced in the newly added paragraph on “Level of PPI contributor’s involvement in this realist review”

 A paragraph on incorporating PPI in realist review has been added to address the formation of the advisory panels.

**How were the advisory groups created? It seems that the advisory groups do not participate equally in the project.**

 To address this query, we have added Table 3 to our methodology. It provides an overview of the advisory group (now labelled advisory panel) including rational for including the specific advisors and their expected roles. Also we have added a summary of advisory group meetings during Step 1 of the review to extended data.

**Did other advisors besides the content and realist advisors play any role in formulating the research questions and deciding on the design of the study?**

 No, only the content and the realist advisors played a role in formulating the research questions and deciding on the design of the study. In the newly added Table 3, we have clarified the roles of all advisors (realist, content, student, PPI and educationalist). Additionally, the roles of advisors are overviewed in the new paragraph included in our introduction.

**What were the participatory methods employed in the project?**

 Participatory methods are not employed in the project. The first author, an experienced speech and language therapist, used supportive communication strategies and techniques to ensure that the PPI contributors with communication impairments:

- Understood important details such as the review’s aims and demands (e.g. level of involvement and time)
- Could actively engage in programme theory refinement (e.g. IPTs presented one at a time and in simpler parts as requires)

**What is the PPI framework that the authors will use to design and monitor the contributions of the 'people with lived experience of stroke acquired communication impairments/PPI advisors'? Referring to the PAOLI framework developed by Charalambous and colleagues in 2023.**

 The authors have included Abrams et al. (2020) who published a series of prompts for researchers and PPI contributors engaging in realist reviews (https://www.spcr. nihr.ac.uk/news/blog/the-role-or-not-of-patients-and-thepublic-in-realist-reviews). The authors have selected this resource given that this protocol is for a realist review.  (Referenced in introduction and methodology)

 In Phase 2 of this project, we will explore other frameworks to capture PPI contributor’s experiences, including the PAOLI framework (Charalambous et al., 2023)

**How will the authors approach the PPI patients in all advisory groups to avoid tokenism, especially those with communication difficulties?**

 The authors are committed to authentic PPI involvement and five of the seven authors are experienced PPI facilitators, additionally six of the seven authors are experienced speech and language therapists who are skilled in using communication techniques and strategies to support the authentic involvement of people with communication impairments. Also, we are adhering to McMenamin et al’s (2021) “top tips”.  We have added additional detail on “Enabling active involvement of PPI advisors in realist review” and provided a sample of the adapted materials used for PPI Advisor 1 in newly added extended data.

**What are the contributions expected from all PPI advisory groups in each phase of the project?**

**Please briefly explain how involving PPI will improve the study's impact and the implementation of the results**

 In phase 1 of this project the PPI advisors have considered, reviewed and contributed to the development of initial programme theories and ensured their validity for people with communication impairments.

 This publication is only related to Phase 1 of the project, i.e., the protocol for the realist review.  All phases of the project are referenced in this publication to provide the reader with context for the realist review. Phases 2, 3 and 4 are not detailed here but, will in due course, be developed in subsequent articles and the contribution of all PPI advisory groups in each phase will be detailed.

**What are the obligations of the 'People with lived experience of stroke acquired communication impairments/PPI advisors' in the research team? This information will ensure transparent documentation of PPI and especially patient advisors’ contributions. It would be useful to include a table showing expected/completed PPI contributions from each advisor group in each project phase.**

 The obligations of the “people with lived experience of stroke acquired communication impairments/PPI advisors” are outlined in the newly added Table 3.

**It would be helpful to provide additional demographic data on the two PPI advisors who have lived experience of stroke and related communication impairments. This data could include information such as the type and severity of the communication impairment, time since the stroke, previous and current employment, marital status, level of education, and psychosocial information. This information would provide the reader with a clearer understanding of the patient advisors' level of involvement within the research team**

 Thank you, brief summary detail on type and severity of communication impairments of our PPI advisors has been added to the methodology section.

 In Phase 1, the authors (experienced speech and language therapists and PPI facilitators) ensured that the supportive communication strategies and techniques employed facilitated authentic involvement of the PPI contributors.

**Please describe how the two PPI advisors who have lived experience of stroke and related communication impairments will be involved in disseminating the project’s outcomes.**

 Advisors will co-create a dissemination plan to share the findings of this research. In collaboration with the PPI advisors, dissemination strategies that will be meaningful to people with communication impairments will be co-developed e.g., co-authorship of research papers; co-presentation at workshops etc. For example, to date one PPI advisor and the lead author have presented their reflections on their experiences of collaborating on a realist review at a Palliative Care Workshop, focused on involving people with communication impairment in research.

**It is suggested that the authors provide a more comprehensive explanation of their approach to supporting PPI patient advisors throughout phase 2 of the project, with particular attention to addressing their communication challenges.**

 Thank you for this recommendation. When reporting on Phase 2 of this project the authors will provide a comprehensive explanation of their approach to supporting PPI patient advisors throughout this phase of the project and addressing their communication needs.
